# Developing and promoting qualitative methods in general practice research: Lessons learnt and strategies convened

**DOI:** 10.1177/14034948221093558

**Published:** 2022-05-21

**Authors:** Kirsti Malterud

**Affiliations:** 1 Department of Global Public Health and Primary Care, University of Bergen, Norway; 2 Department of Public Health, University of Copenhagen, Denmark

**Keywords:** Qualitative methods, general practice, research, science

## Abstract

Fifty years ago, qualitative research methods were unknown in medicine. Biomedicine and the positivist paradigm were universal academic standards. In the late 1980s, however, humanist perspectives emerged as substantial values in general practice. This progress fostered an effort among Nordic general practitioners to find research methods best suited to exploring clinical communication and the doctor-patient relationship. Simultaneously, qualitative methods were promoted internationally in medicine, mostly by social scientists. This article is a personal narrative of the history and impact of Nordic general practitioners customising qualitative methods for the study of clinical practice. I present lessons learnt and strategies convened in developing qualitative methods in this Nordic context. The patient-centred method paved the way for research standards consistent with our clinical ontology. We struggled to develop dialogues that promoted methodological legitimacy among medical colleagues. Methodological standards like rigour and reflexivity became important and contributed to intersubjectivity by sharing the research process. Gradually, our endeavours gained notice. In the last couple of decades, the number of published qualitative studies has increased, though perhaps at the cost of methodological quality. Indeed, there are also indications of a methodological backlash among influential journal editors. Nordic general practitioners have been prominent in developing qualitative methods suitable for cultivation of medical knowledge. Our position of knowing, close to the experiences of the individual patient and the everyday context, is different from that of a social scientist. It offers a unique point of departure for knowledge development that can make an important difference for both patients and doctors.

## Background and approach

*Qualitative research methods* are used to explore the meaning of social and subjective phenomena as experienced by individuals in their natural context [1]. Analysis implies a reflexive process of systematic collection, organisation and interpretation of textual material, mostly from talk or observations [2]. Fifty years ago, when this journal^1^ was founded, qualitative methods were unknown in medical research. For many years, medical research was synonymous with *quantitative methods*, featuring analysis by statistical calculations and objectivity, standardisation, and generalisation as research criteria. Today, the situation is far different. I was among the general practitioners (GPs) who first took up the qualitative research challenge in the Nordic countries. As a participant and stakeholder in the development and promotion of qualitative methods in medical research for the last four decades, I offer this article to share experiences and reflections about lessons learnt and strategies convened along the road.

My presentation is a personal narrative, drawing on my own experiences, reflections and contributions. Still – as in qualitative research more generally – I aim for transferability and relevance beyond my own context [1]. I accentuate the collaborations between Nordic GP researchers, referring to ‘we’ when it comes to joint attitudes and efforts, and ‘I’ when something is more personal. The historical lines I present below have taken place during overlapping periods of time, and my presentation will therefore not follow strictly chronological lines. The reader may find the list of references biased since I present so many of my own publications. This is, however, a frank and honest indication of my own interests and deliveries within this field over the years, which I will not fail to disclose.

## Humanist perspectives entering medical practice and research

In the 1980s, general practice was still a youngster in the family of medical research in the Nordic countries. A few brave GPs had entered research, adopting available methodologies with population studies and practice registrations [3–7]. Their investments of time and effort built the foundations of today’s academic primary health departments, leading to strong research groups with advanced methodologies for epidemiology, randomised controlled trials, and register studies.

During the same period, *humanist perspectives* and *patient-centred methods* gradually gained footholds as substantive values of general practice. Encouraged by international pioneers [8,9], GPs in the Nordic countries embraced these ideas in practice and teaching, emphasising the doctor-patient relationship and clinical communication. This progress also fostered a pursuit of research methodologies appropriate for the exploration of human interaction, subjective experiences and complex processes – phenomena increasingly recognised as indispensable elements of health, illness and health care [10–12].

As a young GP in the late 1970s, I often felt overwhelmed when seeing patients presenting subjective symptoms without objective findings, conditions that later were called medically unexplained symptoms (MUS). Medical school had not prepared me for chronic health problems without diagnosis or treatment. These patients, mostly women, suffered from pain, fatigue and reduced function and were not very popular among GPs. The label “heart-sink patients” attributed the problem to the patient rather than to the complexity of their health issues or to the GP’s lack of understanding [13]. However, some surprising breakthrough experiences of communication gradually encouraged me to embark on a creative journey of developing and implementing ‘key questions’ that were intended to invite the patient to share her knowledge with the GP [14]. After some initial perplexity, I realised that my purpose required methods other than the biomedical strategies that had long been taken for granted in medical research.

## Qualitative research traditions in disciplines beyond medicine

Doing research without statistics was not a new idea in the humanities [15]. Indeed, interpretative traditions are much older than the natural sciences and biomedicine. For centuries, theology, philosophy, history, law, literary research, and to some extent the social sciences, had established *hermeneutic* standards for textual interpretation [16]. Reading a text as an interplay between its individual parts and the whole is crucial, as is the impact of context and self-reflection.

Beginning in the nineteenth century, *phenomenology* was developed as a philosophy to understand subjective experiences and consciousness in human beings [17]. Bracketing one’s own preconceptions is a phenomenological attitude to approach knowledge about a person’s subjective experiences of his or her lifeworld. Phenomenological philosophy seemed especially relevant to psychology, social sciences, and physiotherapy.

*Ethnography* was established in the early twentieth century by social anthropologists conducting cultural studies that involved participant observation [18]. Starting in the 1950s, *social constructionism* evolved as a philosophy considering phenomena as conceived, perceived, and performed by social interaction, leading to an awareness of the particular and even unique rather than the general and universal [19,20]. Meanings, concepts and understanding are generated by discourse, with the diversity of subjectivities more notable than objective facts. Social constructionism was introduced by social scientists and family therapists and has increasingly been adopted as a philosophical platform for qualitative research across disciplines.

These philosophical ideas have evolved as historical building blocks, combining to contribute substantially to what today is called qualitative research. These traditions complement one another and now constitute a strong historical and philosophical foundation for knowing about human meaning and interaction.

## Domains of knowing – assumptions and paradigms

*Ontology* refers to how we understand the world and reality. The stable and predictable ontology of laboratory medicine differs from the unstable and complex ontology of clinical practice, which deals with unique patients in their varied social contexts [12]. *Epistemology* is the analogous concept for development of knowledge about the world and reality. Biomedical research methods with statistical calculations of objectively observed empirical data are tailored to the study of stable ontologies but are not equally appropriate for the study of dynamic ontologies. Consistency between ontology and epistemology is, however, a logical prerequisite for scientific knowledge [21].

In 1959 C. P. Snow, an English novelist and physicist, described the gap between natural science and humanities in the academic world as ‘the two cultures’ [22]. A few years later, the American philosopher Thomas Kuhn presented *scientific paradigms*, which were taken-for-granted fundamental beliefs about the world and knowledge [23]. *Positivism* had been coined by the French philosopher Auguste Comte (1798–1857) for any system that confines itself to the data of experience and excludes a priori or metaphysical speculations [24]. Michel Foucault described the rise of positivism in the 1850s, leading to the development of modern medicine [25]. According to Kuhn, *normal science* is based on ideas holding the status of contemporary universal consensus, typically *the positivist paradigm*. Humanist traditions and qualitative research are conceived as *the interpretative paradigm*, challenging the positivist paradigm as contemporary normal science. (I would, however, comment that all research is interpretative. Since these concepts appear to have been and remain broadly acknowledged, I will nevertheless use them below.)

## Exploring communicative interaction in general practice – motivations and support

Returning to my own research exploring communicative interaction, I realised that questionnaires or practice registrations would not open the gates to the ontology I wanted to understand. My intervention was not suited for a randomised controlled trial. A social anthropologist mentioned something called qualitative methods. Looking into the sparse methodological literature of that time, I felt empty-handed, even helpless. Interview studies and participant observation were the methods of choice, but methods for analysing the resulting data were fuzzy at best [26–28].

I was searching for a design that would allow me to systematically reflect upon particular actions in my own practice – what I said, how the patient answered and the context of this talk. My medical mentors liked the idea and insisted that the team needed a sociologist who knew the tools of the trade. Finally, I conducted my project as an action research study, with audio recordings as empirical data for qualitative analysis of the development and implementation of key questions [14]. The key questions became widely adopted among GP colleagues, indicating their potential as a clinical method for complex challenges.

In the late 1980s, I was not the only one looking for appropriate research methods to study doctor-patient relationships and clinical communication. Similar ideas also evolved among Nordic GP colleagues, triggered by contemporary humanist influences. In Sweden, Carl Edvard Rudebeck studied dialogues in general practice about symptoms and symptom presentations, developing the innovative concept of ‘bodily empathy’ [29]. In Denmark, Inga Lunde interviewed patients about their perceptions of illness experiences, presenting the foundation for shared understanding between GP and patient [30]. Our doctoral dissertations were accepted for defences and degrees, giving us a boost of enthusiasm regarding our future academic prospects. In Bergen, Aarhus and Umeå, additional GP colleagues took up research with qualitative methods, with those in Copenhagen soon following.

In the UK and the USA, qualitative methods in medical research were promoted by social scientists [31,32], with introductory textbooks written by sociologists [27,33–35]. On the Nordic scene, however, the pioneers were medical practitioners whose clinical experiences triggered questions that led towards qualitative research. Only a few had acquired relevant philosophical or methodological competence, which we step by step learnt to catch up in dialogues with research colleagues from other disciplines. The Norwegian social anthropologist Cato Wadel demonstrated how sample size was not a pre-set matter of law [36]. The Swedish sociologist Bo Eneroth offered early foundations for the theory of science and interpretative research [37]. The Danish psychologist Steinar Kvale became especially influential for general practice research [38]. Inspired by phenomenology and postmodernism, Kvale’s ability to present and implement complicated methodological questions was great. These cross-disciplinary connections inspired us as GPs – at the same time, though, they gave rise to reflections and objections regarding ontology and epistemology.

## Nordic GPs – vanguards for qualitative research in medicine

‘Travelling ideas’ have been suggested to contribute to institutionalisation [39], and the humanist perspectives crossing the Atlantic and North Sea had a powerful impact on general practice in Europe at this time. These ideas also encouraged corresponding research in Nordic clinical practice, as demonstrated by two chapters about qualitative projects under the heading ‘Methods for better understanding of patients’ in a 1988 general practice research textbook [40]. From 1991–1998, a Nordic task force arranged four seminars about qualitative research in general practice [41]. The participants were Nordic GP researchers with experiences from qualitative projects. Of the 42 people who participated in at least one of these seminars, 24 later earned doctoral degrees. These Nordic seminars became a unique meeting place for negotiations about methodology on general practice premises.

Our common drive was to develop medical knowledge that could make a difference for both patients and GPs – a more specific ambition than simply adoption the social sciences as a blueprint for our methodological pursuits. Some of us chose designs so complex that we today would advise against them. Supported by various theoretical perspectives, we studied ourselves or our colleagues interacting with patients in audio- or video-taped consultations with or without interventions [14,29,42–44]. Others applied more established qualitative methods, especially semi-structured interviews [45–47]. Later, participant observation and focus group studies added to the diversity of designs that could provide the best possible match with the research questions [48,49]. In these early years I was, for example, involved in interview studies about women’s experiences of pelvic examinations [50] or about symptom experiences of patients with chronic fatigue syndrome [51]. Other studies were based on analysis of audiotaped recordings from consultations where patients’ self-assessed health resources were explored [52] or where analysis highlighted the GP’s use of talk as a medical tool [43].

Ideas growing in the Nordic group of GPs travelled across national borders, contributing to the early, if humble, institutionalisation of qualitative methods in medical research in these countries [39,41]. We shared inspirations, experiences and reflections and approached epistemological advancements customised for general practice ontology. Strategies were digested, elaborated and summarised in introductory textbooks written in Nordic languages and addressed to novice medical researchers [53,54]. Concepts and procedures were established, sometimes in hindsight when we noticed corresponding ideas from somewhere else. *Transparency and intersubjectivity* were established as crucial perspectives to understand and communicate the research process [55]. This also encouraged our awareness of *reflexivity* – an active attitude the researcher must seek out and maintain, recognising the role of the researcher and the social context in which versions of reality are interpreted and situated with positions and perspectives [2,15,56,57]. We learnt to draw attention to the impact of the researcher’s *preconceptions* (experiences, motivation, ideas, hypotheses) ahead of data collection and analysis [16] and slowly started supporting our interpretations with *theoretical perspectives* [58]. Such perspectives became especially important in studies where the researchers were also experienced within the field of the study, such as a focus group study where GPs reflected upon experiences of talking about alcohol use with patients who did not themselves initiate the topic [59]. In another study, theoretical perspectives about shame enhanced the focused analysis of videotaped consultations dealing with lifestyle issues [60].

## Reception of qualitative research methods in the biomedical culture

As pioneers and novices introducing qualitative research to our medical colleagues, we were often met with considerable scepticism. This also happened in friendly settings and among GP researchers. The harshest critics seldom took the effort to familiarise themselves with methodological traditions and standards outside their own environment. Similar comments were repeatedly heard from reviewers in medical journals, who took for granted that the positivist paradigm and biomedical methods represented universal scientific truth.

Representative and large samples for extrapolation of population prevalences, control groups for comparison of effect measures, reliability for repeated standardisation checks, or generalisability for unlimited validity were often demanded by reviewers, irrespective of research aim or method. All these issues are appropriate tools for the analysis of numerical data with statistical calculations about epidemiological questions. However, purposive samples for adequate content validity (which were never intended for effect studies), variability for nuances and potential contradictions (rather than biases) and transferability of concepts and interpretations beyond the study context are considerably more appropriate for the analysis of textual data [1].

Qualitative researchers often responded with separatism, insisting on incommensurable dissimilarities, especially when demands were raised with reference to the supremacy of positivist concepts and procedures. Encountering such expectations and demands, we might become tired or annoyed, to the extent that some resorted to dismissing all traditional research criteria. Others tried to translate central methodological concepts from quantitative to qualitative research. Nicholas Mays and Catherine Pope maintained that qualitative research could be assessed with reference to the same broad criteria as quantitative research, albeit used in a different way that highlights validity and relevance [61]. Yvonna S. Lincoln and Egon Guba suggested alternative criteria, comparing credibility with internal validity, confirmability with objectivity, and transferability with generalisability [34]. Katarina Hamberg and colleagues pursued this line of thought when discussing the potential of translating different established concepts and criteria [62]. Kvale successfully reintroduced appropriate aspects of validity that are well suited to qualitative research. [63]. My own contributions along these lines included a proposal of relevance, validity and reflexivity as overall metacriteria [1].

## Addressing and advocating legitimacy

In presenting our work to colleagues from biomedical research, we had dual aims: first, we wanted to argue for and demonstrate important aspects of the specific knowledge base in general practice. Second, we aimed to have an impact on medical research in general by demonstrating methods that had the capacity to develop evidence about distinctive elements of clinical practice. Hence, promoting *legitimacy* for qualitative methods in medical research became a strong motivation. We had to understand and explain what we were doing and to develop capacities to reflect upon contexts, elaborate methods and share processes to include colleagues without previous knowledge of the field. Successful dialogues seemed to require carefully considered rhetoric, with appropriate and sometimes brave communication of relevant arguments, preferably presented in a calm and patient manner. The latter remarks may seem irrelevant or exaggerated today, but sometimes the atmosphere could be genuinely hostile and even degrading, indicating a strong antagonism.

A principal line of logic dealt with balancing *differences and similarities* between qualitative and quantitative methods. In methodological textbooks, qualitative methods were often presented as opposing quantitative methods, with columns of dichotomous scientific values. Moreover, the qualitative columns could boast moral virtues (closeness, holism, empowerment, humaneness) that contrasted with the characteristics of quantitative research (distanced, objective, technical). Such attitudes seemed to increase the polarisation between positions, neglecting our overall joint efforts to develop medical knowledge. Sometimes, this dichotomous position could also be perceived as a call for replacing biomedical research with qualitative methods. An important counterargument was therefore to emphasise quantitative and qualitative research methods as *complementary*, endorsing the most appropriate match between methodology and the specific research question. Quantitative methods were superior for measurement of effects and prevalences, while qualitative methods were outstanding to explore subjective experiences and human interactions. None was universally excellent.

A worthwhile approach was to present *scientific traditions and assumptions* for developing trustworthy evidence within the interpretative paradigm. Using the history and theory of science, we tried to make sense of the negative arguments we met. Some were adequate, especially when it came to transparency. The process of qualitative analysis was often presented as implicitly leaving a mysterious black box of interpretations. Sometimes, our philosophical pursuits became inflated, blurring the basic connection to clinical knowledge. Friendly comments from colleagues would usually help us get back on track. These efforts became an interesting and useful journey, convincing several of us that focusing on the overall similarities between qualitative and quantitative research might constitute a constructive platform for dialogue, unless we compromised substantial logical differences in our operationalisation of methods.

A major shift in legitimacy and respect for qualitative methods in medical research was experienced after the publication of two articles in *The Lancet* in 2001 [1,12], including the following statement [1] p 483

Qualitative research methods are founded on an understanding of research as a systematic and reflective process for development of knowledge that can somehow be contested and shared, implying ambitions of transferability beyond the study setting.

Although this definition was crafted specifically for qualitative methods, few audiences – regardless of research tradition – were willing or able to dismiss this definition of research. By establishing a joint position among medical researchers, I felt it was easier to challenge the universal supremacy of concepts and procedures developed for numerical analysis and to argue why other methods would be more appropriate for the thoughtful, nuanced, and above all scientific analysis of text and meaning.

## Research standards and rigour

Qualitative research was persistently denounced as ill-founded opinions and speculations, as an easy choice for those who lacked the capability for statistics. Realising the power, monopoly and taken-for-granted nature of the positivist paradigm, we learnt – often the hard way – that *methodological rigour* was necessary to be understood and taken seriously among our medical research colleagues. As a crucial point of departure, we therefore needed to ensure that we knew what we were doing and were sufficiently clear to explain specifically why and how [55,62]. In hindsight, I believe this special emphasis on methodological transparency and intersubjectivity among Nordic GP researchers also emerged as a strategy to counterbalance our claims of the overall similarities between qualitative and quantitative methods. Mutual respect could only be achieved when we were able to demonstrate transparently that systematic, reflexive approaches were substantial elements of our research.

This strategy was never explicitly voiced as such. From around the turn of the millennium, it took place in several arenas, becoming elaborated and sometimes published. In the *Lancet* article, I also presented a checklist for assessing how relevance, validity and reflexivity were attended to in publications reporting qualitative research [1]. Twenty years later, I find this checklist far too detailed. At that time, however, it functioned as a concrete roadmap for systematic approaches to qualitative research, illustrating that it was possible to distinguish good from questionable quality. The metacriteria and checklist also visualised some epistemological values, referring to a social constructionist position by emphasising the impact of the researcher and the context [19]. Furthermore, rigour was indicated by highlighting transparent and systematic approaches.

Today, there are better checklists available for the evaluation of qualitative research, which are now developed according to rigorous validation procedures. Qualitative methodology has rapidly evolved on a global scale in the last two decades, with most contributions still coming from social scientists. Confronted with an apparently uniform methodological consensus in biomedical research, it was not always easy to explain and defend the broad methodological diversity among qualitative researchers. The mixture of positions and opinions also revealed the existence of a vital research community with a clear capacity for growth and development. Medical researchers have been learning along the road, developing our thoughts and arguments. My own introductory textbook is now appearing in its fourth edition, significantly elaborated from the first one 25 years ago. During these years, I have learnt a lot from prolific interdisciplinary collaborations. Still, it has become increasingly clear to me that the GP researcher better accommodates his or her academic potential by elaborating the epistemological challenges and opportunities evolving from the clinical ontology than trying to be a sociologist.

Gradually, several of the initial Nordic GP pioneers advanced to become senior academics, serving as supervisors for PhD students and reviewers for funding sources and medical journals where qualitative research slowly became incorporated. We were writing and revising textbooks, teaching and presenting qualitative methods, and conducting empirical studies. When we were reviewed, we took the opportunity and expended the effort to respond to and sometimes reject the objections. From these positions, we established certain methodological standards.

These standards were not always the same in journals from other academic disciplines or regions. In the social sciences, qualitative s evolved faster than in medicine. Qualitative research, while rarely mainstream in the social sciences, still appeared far less alien than in the biomedical domain. Among social anthropologists, on the other hand, issues of method were more often taken for granted. Medical researchers were questioned why we always had to explicate our methodological assumptions, as had become our habit. In hindsight, I admit that we probably overstressed method, and numerous comments from reviewers led to more modest efforts. Still, I believe that this strategy added to several examples and arguments for why research methods must be tailored to the discipline and the research question, both ontologically and epistemologically.

Another interdisciplinary challenge is related to the different traditions and ambitions for use of theory in qualitative studies across academic disciplines. When theories are conceived by researchers from the humanities or the social sciences as a mandatory point of departure or an ambition for analytic development in a qualitative study, the medical researcher more often applies theoretical perspectives to sharpen the aim and understanding or to situate an empirical study. Such differences reflect ontological and epistemological variations across disciplines rather than an inherent academic orders of excellence [58]. Drawing on a broad range of positive cross-disciplinary collaborations. I have, unfortunately, also experienced misunderstandings, conflicts and rivalry. There is still work to be done within qualitative research to pay the distinctiveness of disciplines duly mutual respect.

## Promotion, implementation and inflation

To us as pioneers within qualitative research, it was essential to develop knowledge relevant for general practice and also to establish mutual dialogues with our biomedical peers. The first decade of the millennium demonstrated how our endeavours to promote qualitative methods as appropriate tools for medical research were attended to. Quite rapidly, the number of published articles reporting medical qualitative studies began increasing. Journals for general practice research extended their permitted standard maximum number of words from 2500 to 4400 for articles reporting qualitative studies. Qualitative research was implemented not only as appendixes to quantitative studies but as self-sufficient, independent scholarship.

However, enthusiastic students were not always supported by supervisors with adequate methodological competence. Senior biomedical researchers left their footprints in research applications or manuscripts, either by taking positivist standards for granted (‘To be absolutely sure to eliminate bias, we set up and compared independent coding’), or on the other hand being naïve or superficial planning a study (‘We will do a focus group study, details will follow’) or about systematic methods for analysis (‘We discussed the data and categories emerged’). Serving as reviewers and opponents, we noticed a trend of descriptive studies with broad aims reproduced already existing knowledge or trivial issues in no need of research (‘The participants agreed that it was important to be taken seriously by the doctor’). In such cases, a basic understanding of the interpretative paradigm would have been helpful. Similar attitudes were demonstrated among reviewers and journal editors, who suggested quotations to be decontextualised in boxes separate from the text, as if they were independent findings that could be presented in a table.

Several journals were excited about first-person accounts and published descriptive articles with limited information about interpretation and analysis. Asking ‘Why are interview studies so boring to read?’, Kvale discussed this phenomenon [38], referring to a trend of qualitative articles characterised by profuse quotations and insubstantial findings. *Mixed methods* studies also became increasingly popular, but articles demonstrating an appropriate analysis with integration of the applied methods are still exceptional. As Nordic GP researcher colleagues discussing these issues, we noticed a paradoxical challenge of this period: Qualitative methods had evolved, gained increased recognition and were being broadly implemented, while sufficient methodological competence was not always present among supervisors, reviewers or editors [64].

## Methodological progress, elaboration and diversity

After convening strategies for presentation and promotion of qualitative methods in the previous decades, implementation and development became less tense in the 2010s. GP researchers seemed more confidently affirmed in the interpretative paradigm, realising the strengths of these methods to reveal clinical knowledge. Multi-method doctoral dissertations, with one or two of the articles being qualitative, became quite common. Debates about specific methodological issues such as *sample size* could now be better anchored in shared epistemological understanding [65]. While *analysis* had previously often been an impressionist concern combining elements from different approaches, several methods with feasible procedures – mostly from the social and health sciences – were available and implemented. Thematic analysis was often preferred among Nordic GP researchers [66–68], and phenomenological analysis was also a popular choice [69–71]. However, the North American tendency to use phenomenology as a very broad category to cover any qualitative method was sometimes observable also among Nordic GP researchers.

The increasing body of publications and methodology on a trans-national scale demonstrated a growing diversity among qualitative researchers across disciplines, also within medicine and health research. Positions, standards and opinions could sometimes be conflicting, leading to discourses about the one and only orthodox and acceptable way of doing qualitative research. This was probably also a response to the earlier impressionist period, during which many researchers, lacking sufficient professional guidance, idolised the freedom of customising their personal approaches to design and analysis. Among Nordic GP qualitative researchers there was also a diversity in approaches, though never leading to strong oppositions or conflicts. Qualitative research could no longer be regarded as a toddler in the research family, and methodological requirements became sharper. Purely descriptive studies were more often seen as trivial. Attention was drawn to the creative value of subjectivity, striving for more complex analysis supported by theoretical perspectives with consequently more original findings [58,72]. Senior qualitative researchers were paying increasing attention to the meaning of subjectivity, reflexivity and interpretation, likely as a result of improving external recognition [2,15]. The researcher’s assignment was expected to be something other and more than being a microphone stand for the participants, simply reporting what was said. Sometimes, an orthodox devotion to procedures and philosophies could get the upper hand, accentuating procedures and checklists at the expense of interpretation and findings. Exaggerating method in this way has been called ‘methodolatry’ *–* a position entailing the risk of instrumentalisation [73].

## Where are we now – and where do we go from here?

The last decades have fostered development and progress for qualitative research methods in general, also within Nordic GP research. Many journal editors and reviewers now welcome qualitative studies and are better able to offer appropriate assessments of submitted articles, with a broad range of study aims, research designs, theoretical perspectives and methods for analysis. Interview studies (individual or focus group) with thematic analysis are probably the most common approaches. But there are also creative examples of (among others) observational studies, ethnographies, Internet-based studies, studies of retrospectively or proactively written texts or action research studies. Relevant choices of analysis methods such as narrative analysis, discourse analysis or different varieties of phenomenology enhance the outcome of original knowledge, as can also intriguing theoretical perspectives.

While the *Scandinavian Journal of Public Health* has a fine record in this regard, other journals are demonstrating attitudes suggesting an epistemological backlash. The *British Medical Journal* (BMJ) had previously pioneered qualitative research in medicine, regularly publishing empirical studies and methodological education. In 2016, the journal announced a change of editorial policy that made qualitative studies an extremely low priority, ‘since they are not as widely accessed, downloaded, or cited as other research’ [74]. Seventy-six senior academics from 11 countries urged the BMJ editors to reconsider this policy and challenged the journal to develop a proactive, scholarly and pluralist approach to research that aligns with its stated mission [74]. The editors responded by confirming their opinion, aiming to ‘publish studies with more definitive – not exploratory – research questions that are relevant to an international audience and that are most likely to change clinical practice and help doctors make better decisions’ [75]. These arguments reinforced an impression of limited methodological proficiency at one of the world’s most esteemed medical journals.

The BMJ case emphasises the urgent need for qualitative researchers to enhance and exhibit their capacity to deliver medical knowledge of high relevance and methodological quality. As a member of a research community of GPs devoted to the ongoing development of methodology, I have experienced the joy of being able to contribute with original knowledge for practice. Nordic GPs have been able to customise qualitative methods in line with the ontology of our medical discipline. We were forerunners in developing and implementing qualitative methods in medicine, leading to research knowledge that is profoundly relevant for clinical practice and thus for the patients that all doctors are devoted to serve. Our position of knowing, close to the experiences of particular patients and their everyday contexts, is a unique point of departure for development of knowledge that can make a difference for both patients and doctors. Demonstrating that evidence from research with qualitative methods may have such a potential, is the best argument for further implementation in medicine.

## Notes

1 Originally: Scandinavian Journal of Social Medicine, since 1999: Scandinavian Journal of Public Health.
